# Progress in Ophthalmology

**Published:** 1899-10-21

**Authors:** 


### PROGRESS IN OPHTHALMOLOGY.

(*Continued from page* 33.)

**Iritis.**—Dr. Percy Dunn,[5] in his paper on iritis, says that the causes of iritis may be cut down to two, namely, rheumatism and syphilis, and of these two the rheumatic is now the most common. As far as syphilitic iritis is concerned, he thinks, as time progresses, it will be less and less frequently seen, from the fact that the treatment of primary syphilis is now so much more scientific and thorough than in earlier days. Furthermore, it is only rarely now that the cases of double syphilitic iritis are met with, the explanation being that patients come early under efficient treatment before the virus has had time to expend itself on both eyes. He points out that there is one redeeming feature in the syphilitic variety of iritis, namely, the almost complete absence that it shows of any tendency to relapse. As to the pain in these cases, it is true to remark that this is a variable symptom; but it is not correct to say that the pain in specific iritis is commonly insignificant in degree. Experience teaches that the pain is often most acute, imperatively requiring active treatment for its relief. Again, the rule is for it not to recur after its relief has been once effectually secured. As to rheumatic iritis, it is not always possible, he says, to obtain a personal history of rheumatism, but frequently he finds patients are able to admit to a history of gout or rheumatism in their parents. The unfavourable feature is the tendency which rheumatic iritis shows to recurrence. Thus it is that rheumatic iritis, by its frequent recurrence, may prove very harmful in its effects. In time posterior synechiæ form, the iris structure degenerates, the capsule of the lens becomes thickened in the pupillary area, and the eye practically useless for visual purposes. As to the treatment, he points out that from time to time the error has been made of treating inadvertently rheumatic iritis as conjunctivitis, and of applying astringent lotions, either of sulphate of zinc or even nitrate of silver. In syphilitic iritis he deprecates the pushing of the mercurial treatment to the point of salivation. As to the local treatment, he says beyond the instillation of atropin it is practically nothing, unless in the cases where there is great pain, and that this can easily be subdued by local depletion; one or two leeches to the temple will quickly relieve the pain. While on the subject of pain he refers to the treatment of that met with in the rheumatic variety; local depletion in these cases is less indicated. In acute syphilitic iritis the pain is caused mainly by the intense vascularity of the ocular tissues; on the other hand, in rheumatic iritis it appears to be more of a neurosis, comparable to that met with in other rheumatic affections, consequently, in the relief of the nocturnal pain of rheumatic iritis, nothing succeeds so well as the application of dry heat. Some surgeons recommend moist heat, but he thinks the use of moist heat in eye surgery is best confined to the affections of the cornea, and his particular form of dry heat is the old-fashioned bran poultice.

In rheumatic, as in syphilitic, iritis, it is, of course, most important that the patient should, as soon as possible, be brought under the influence of constitutional remedies. As to the length of time the atropin should be continued after the evidences of inflammation have subsided, his opinion is a month in all cases. In specific iritis the first indication of improvement noticed is the restoration of the transparency of the aqueous humour; in rheumatic the first indication is commencing subsidence in the vascularity of the globe. He also deprecates the keeping of the patient in a dark room; all that is requisite is to order a shade to be kept over the affected eye, supporting a large pad of absorbent wool. By this means complete rest and warmth are secured for the affected organ, and with the pupil well under atropin no harm can ensue from any light to which the fellow eye may be exposed. Another point he raises is this: Is it any use instilling atropin in a case of chronic relapsing iritis in which the pupil is bound down by posterior synechiæ? and he is very emphatic in reply. There is no doubt the drug is of use in these cases. It acts as a sedative, even if it cannot cause dilatation of the pupil. Atropin, therefore, should be instilled in chronic recurrent cases just as frequently as if the maximum benefit from its employment were being obtained. Its sedative effects are often most marked, and the patients are not slow to express the relief which they derive from its use.

**Euphtalmine.**—Treacher Collins,[6] in describing euphtalmine, says: A drug which will quickly dilate the pupil, without disturbing the power of the accommodation or producing any unpleasant toxic symptoms or local irritation, and whose effect quickly passes off, is likely to prove of great use in ophthalmoscopic examinations. Euphtalmine apparently possesses all these estimable qualities; it is a synthetic preparation, its full chemical name being the hydrochloride of n-methyl-vinyl-diacetone-alkanine. Vossius described its action in 1897. Two or three drops of a 2 per cent. solution produce full dilatation of the pupil in 20 to 30 minutes, and its effect entirely disappears in from two to three hours. A 5 to 10 per cent. solution very slightly influences the accommodation, not sufficient to hinder near work; its effect only lasts a very short time.

**Quinine Amaurosis.**—Dr. Zanotti[7] reports a case of quinine poisoning. The patient, a man of 38, swallowed 12 grains of quinine for a wager. Within half an hour he lost consciousness, and was violently convulsed. On recovering consciousness some hours later, he complained of "flashes before the eyes," loud noises like thunder in the ears, and people moving in the room appeared like moving shadows. Medical aid was not sought till 40 hours later; amaurosis was then complete, the pupils widely dilated and immobile, hearing quite gone on the right side and much impaired on the left; patient in a state of hebetude, which persisted six or seven days.

The amaurosis remained complete for 10 days, and then the vision gradually returned by almost imperceptible gradations. A year afterwards his vision was three-fifths, fields greatly contracted, achromatopsia for green, dyschromatopsia for the other colours, night blindness. Ophthalmoscopic examination showed white discs, filiform vessels, little hazy white spots round discs and maculæ, probably choroido-retinal changes following hæmorrhages.

[5] Clin. Jour. July 19, 1899. [6] Practitioner, Aug., 1899. [7] Jour. of Tropical Med., July 1899.

